# A Standardized Mouse Model for Wound Infection with *Pseudomonas aeruginosa*

**DOI:** 10.3390/ijms252111773

**Published:** 2024-11-01

**Authors:** Jie Hou, Qian Wu, Rongrong Xiong, Pradeep K. Malakar, Yongheng Zhu, Yong Zhao, Zhaohuan Zhang

**Affiliations:** 1 College of Food Science and Technology, Shanghai Ocean University, 999# Hu Cheng Huan Road, Shanghai 201306, China; hj990918@163.com (J.H.); 13540641547@163.com (Q.W.); rongr_x@163.com (R.X.); pkmalakar@shou.edu.cn (P.K.M.); yh-zhu@shou.edu.cn (Y.Z.); 2 International Research Center for Food and Health, Shanghai Ocean University, 999# Hu Cheng Huan Road, Shanghai 201306, China; 3 Laboratory of Quality & Safety Risk Assessment for Aquatic Products on Storage and Preservation (Shanghai), Ministry of Agriculture and Rural Affairs, 999# Hu Cheng Huan Road, Shanghai 201306, China; 4 Shanghai Engineering Research Center of Aquatic-Product Processing & Preservation, 999# Hu Cheng Huan Road, Shanghai 201306, China

**Keywords:** *Pseudomonas aeruginosa*, standardized mouse model, wound infection, immune response, histopathology

## Abstract

*Pseudomonas aeruginosa* is a highly drug-resistant pathogen known to impair wound healing and provoke inflammatory responses, potentially leading to immune dysregulation. This study aimed to systematically investigate the immune response mechanisms mediated by cytokines following *P. aeruginosa* infection through the development of a standardized wound model. Kunming mice were selected as experimental subjects and given 8 mm diameter lesions on their backs and inoculated with standard strains PAO1 and PA14. The key parameters assessed included changes in body weight, wound redness and swelling, bacterial dynamics, protein content in wound tissues, immune responses, and pathological alterations. The results demonstrated that pathogen invasion significantly inhibited wound healing, with healing rates in the infected groups (87.5 ± 6.3% and 77.1 ± 3.6%) being notably lower than those in the uninfected control group. *P. aeruginosa* persisted in the wounds for up to 12 days, with bacterial loads decreasing from 8 log to 2 log. Additionally, there was a marked reduction in the protein content of the wound tissue and an increase in the expression levels of inflammatory factors such as IL-1β and TNF-α. The thickness of granulation tissue and the number of neovessels were significantly lower compared to the uninfected control group. This study establishes a standardized paradigm for creating a mouse model of *P. aeruginosa* infection in wounds, emphasizing the importance of appropriate mouse strains, uniform wound preparation methods, and moderate inoculation doses for reliable and accurate experimental results. These elements will facilitate the assessment of changes across six key indicators post-infection, providing a foundational data set and technical support for future mechanistic investigations of *P. aeruginosa* infection and the development of targeted antimicrobial strategies.

## 1. Introduction

*Pseudomonas aeruginosa* is an opportunistic pathogen characterized by its highly adaptable and diverse phenotypic traits [1,2]. This bacterium is commonly encountered in various types of skin injuries, including chronic, diabetic, and burn wounds. Furthermore, the World Health Organization has categorized *P. aeruginosa* as a critical multidrug-resistant pathogen within the ESKAPE group [3], which exacerbates the challenges associated with the clinical management of wound infections caused by this organism [4]. The clinical risks posed by *P. aeruginosa* in wound infections are substantial. First, *P. aeruginosa*, as a prevalent wound infection bacterium [5], forms bacterial aggregates that enhance its adherence to the wound [6]. Second, it damages host tissues and immune cells by secreting a variety of enzymes and toxins [7], including elastase, endotoxin lipopolysaccharide, and exotoxin A, leading to local tissue necrosis and an exacerbated inflammatory response, thereby impeding the wound healing process. Third, the biofilm formed by *P. aeruginosa* during infection [8] not only increases bacterial resistance but also hinders effective bacteria clearance by antibiotics and the immune system [9], contributing to the scarcity of effective therapeutic strategies for *P. aeruginosa* infection in wounds. Consequently, a comprehensive investigation into the phenomenon of redness, swelling, pus secretion, and wound edge ulceration caused by *P. aeruginosa* is of significant practical importance.

The development of standardized animal models provides a robust foundation for investigating the pathogenicity of *P. aeruginosa* infections in cutaneous damages, formulating effective prevention strategies, and evaluating the therapeutic efficacy. Currently, numerous studies employ animal models to examine *P. aeruginosa* wound infections. Saima et al. [10] inoculated SD male rats with a *P. aeruginosa* suspension at a concentration of 1 × 10^−9^ CFU/mL in 2 cm diameter lesions, assessing infection impact through wound observation and histopathological analysis. The findings indicated that untreated wounds had only approximately a 30% reduction in size on day 23, alongside significant *P. aeruginosa* residue, a pronounced inflammatory response, and a lack of neovascularization. Huang et al. [11] established a wound model for drug-resistant *P. aeruginosa* (CI-I) by applying a CI-I bacterial suspension at a concentration of 1 × 10^−9^ CFU/mL to 8 mm diameter lesions on the backs of male Kunming mice, noting yellow exudate and pus by day 7, with persistently elevated inflammatory factors observed on day 14. Chelsea et al. [12] created a chronic infection model by infecting 6 mm diameter lesions on the backs of Balb/c mice with a 1 × 10^−6^ CFU/mL *P. aeruginosa* suspension. Laulund et al. [13] utilized alginate beads containing PAO1 in burn wounds on the backs of Balb/c mice, euthanizing the mice on day 8 to collect tissue samples for bacterial quantification and cytokine analysis; they found high bacterial loads and increased IL-1β levels in wounds treated with PBS alone. Chen et al. [14] simulated bacterial infection in diabetic wounds by inoculating 1 × 10^−6^ CFU/mL of *P. aeruginosa* into 4 mm diameter lesions on diabetic mice, with bacterial loads remaining consistent on day 9. While these models elucidate certain effects of *P. aeruginosa* on wound healing, comparing them presents significant challenges that complicate infection severity assessments. The lack of standardized models for *P. aeruginosa* infection exacerbates these issues, hindering consistent results analysis and limiting the applicability of these models in elucidating the mechanisms underlying *P. aeruginosa* wound infections and potential treatment avenues.

This study aimed to establish a standardized paradigm and provide recommendations for animal models of *Pseudomonas aeruginosa* infection in wounds, with the goal of enhancing pathogen invasion management. Kunming mice were utilized as experimental subjects, with an 8 mm wound created on their backs. The wound was then inoculated with a 20 μL aliquot of a 1 × 10^−8^ CFU bacterial solution. Wound changes were assessed by measuring physiological indices in mice, capturing images of the wounds, and recording wound diameters. Additionally, colony counting and Giemsa staining were employed to observe variations in *P. aeruginosa* presence in the wounds. Inflammatory factors, including TNF-α, IL-1β, and TGF-β, were measured and analyzed alongside immunohistochemical staining to evaluate inflammatory expression and the presence of neoplastic cells. Furthermore, protein detection and histopathological staining were utilized to examine the infection within wound tissue. We anticipate that the standardized paradigm developed in this study will provide a robust scientific foundation for elucidating the mechanisms of *P. aeruginosa* wound infection and advancing therapeutic interventions.

## 2. Results

### 2.1. Effect of P. aeruginosa Infection on Body Weight and Wound Healing Rate in Mice

**Figure 1:**
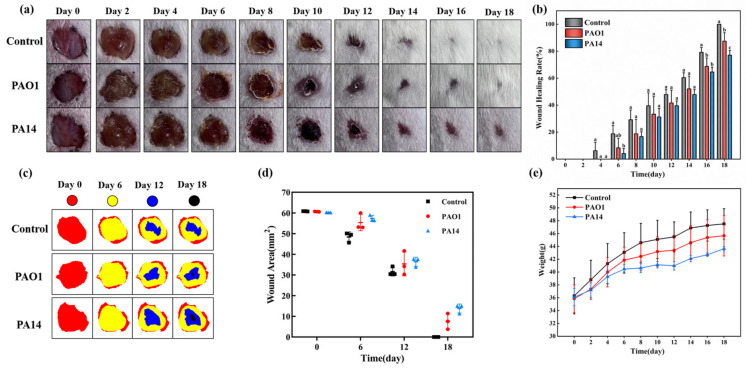
The body weight changes and wound observations of mice in control, PAO1-infected, and PA14-infected groups (control group wound was not infected with *P. aeruginosa*, PAO1 group wound was infected with *P. aeruginosa* PAO1, and PA14 group wound was infected with *P. aeruginosa* PA14). (**a**) Representative pictures of wounds at different time points of mice in each group after surgery. (**b**) Wound healing rates of mice in each group every 2 days after surgery. (**c**) Schematic diagrams of wound areas during the healing process of wounds of mice on days 0, 6, 12, and 18 after surgery. (**d**) Relative wound areas of mice in each group in (**c**). (**e**) Changes in body weight of mice in each group every 2 days after surgery. Statistically significant differences are denoted by different letters (*p* < 0.05).

Wounds were categorized based on the number of damaged skin layers and the size of the affected area, classifying them as superficial, partial-thickness, or full-thickness injuries [15]. In this study, skin wounds on the backs of mice were photographed every 2 days, and the results are shown in Figure 1a. Observations revealed that while impairments across all groups exhibited a trend of gradual healing, significant differences in healing rates were noted. Two days post-wound modeling, wounds in the uninfected control group, as well as those infected with PAO1 and PA14, and the PA14-infected group had formed crusts. However, redness and swelling were evident in the PAO1 and PA14 groups. By day 8, the control group had initiated hair regrowth and experienced scab removal, a process also observed in the PAO1-infected group. In contrast, the PA14-infected group continued to show pronounced redness and swelling. By day 12, slight hair growth was noted around the impairment sites in the PAO1-infected group, while desquamation of the scabs occurred across all groups. On day 18, the wounds of the control group were fully healed with nearly complete hair regrowth. In the PAO1-infected group, substantial hair growth was observed, though a minor open wound persisted. Conversely, the PA14-infected group demonstrated incomplete wound repair and minimal hair regrowth. Figure 1c illustrates the wound-healing process in mice. Preliminary observations suggested that *P. aeruginosa* infection negatively impacted wound healing compared to the control group. To more accurately assess wound infection, further quantitative analysis was performed on the back impairments of mice, and the results are presented in Figure 1b,d. The findings indicated that wounds of the control group began to heal on day 4 post-wound modeling, whereas wounds of the infected groups commenced healing on day 6. The healing rates for the infected groups were 8.3 ± 7.2% and 4.1 ± 3.6%, significantly lower than the control group’s rate of 18.8 ± 6.3%. Statistical analysis revealed significant differences between the PA14-infected group and both the control and PAO1-infected groups (*p* < 0.05). Throughout the healing cycle, the healing rates for both infected groups remained consistently lower than those of the control group. On day 18, the control group had achieved a 100% healing rate, while the PAO1-infected and PA14-infected groups had healing rates of 87.5 ± 6.3% and 77.1 ± 3.6%, respectively (*p* < 0.05). Analyses of wound areas, as depicted in Figure 1d, showed that by day 18, the control group had no remaining wound area (0 mm^2^), whereas the PAO1-infected and PA14-infected groups retained approximately 7.6 mm^2^ and 13.9 mm^2^ of wound area, respectively. This disparity in wound healing was likely due to the heightened inflammatory response induced by *P. aeruginosa* infection, which impeded the rate of skin injury repair.

Body weight serves as a crucial physiological indicator of mouse health. Figure 1e illustrates the changes in body weight across different groups of mice throughout the healing cycle, revealing a general trend of increasing body weight in all groups. At the onset of wound modeling, the initial body weights of mice across the three groups were comparable. However, as the study progressed, mice in the two infected groups exhibited a slower rate of weight gain compared to the control group, especially the mice in the PA14-infected group, which showed the slowest weight gain. These observations suggest that *P. aeruginosa* infection may influence body weight changes in mice to some extent, although all groups displayed an overall increasing trend in body weight.

### 2.2. Changes of P. aeruginosa in Wounds over Time

**Figure 2:**
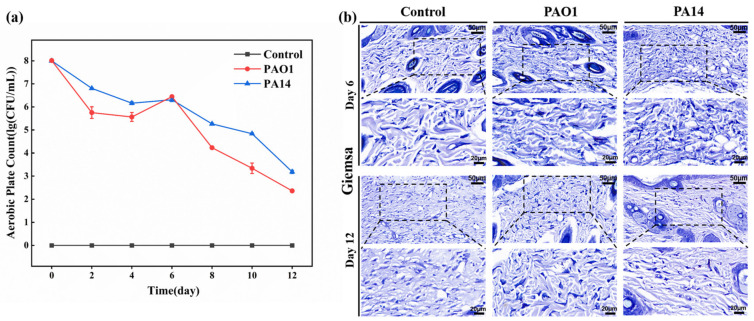
Changes of *P. aeruginosa* in postoperative wounds of control, PAO1-infected, and PA14-infected mice. (**a**) Counts of *P. aeruginosa* colonies in each group every 2 days postoperatively. (**b**) Giemsa staining of wound tissues of mice on days 6 and 12 postoperatively (upper magnification 30×, lower magnification 60×). Data are expressed as mean ± standard deviation (*n* = 3).

To investigate the dynamics of *P. aeruginosa* in infected wounds and further assess the severity of infection, a quantitative analysis was performed using the plate counting method. As shown in Figure 2a, on day 4 post-wound modeling, bacterial counts in both the PAO1-infected and PA14-infected groups decreased compared to day 0. However, substantial levels of *P. aeruginosa* remained detectable, with counts of 6.1 log CFU/mL and 5.5 log CFU/mL, respectively. By day 6, *P. aeruginosa* counts increased by 0.2 log CFU/mL in the PAO1 group and 0.9 log CFU/mL in the PA14 group relative to day 4.

Giemsa staining results (Figure 2b) confirmed the presence of a higher number of *P. aeruginosa* in the wounds of mice from both infected groups. Despite this, there was a general trend of decreasing bacterial counts over the test cycle in the PAO1-infected and PA14-infected groups. In contrast, no *P. aeruginosa* was detected in the wounds of the uninfected control group (Figure 2a,b). In conclusion, *P. aeruginosa* can persist in infected wounds for extended periods, contributing to ongoing erythema and pus production.

### 2.3. Effect of P. aeruginosa Infection on Wound Tissue Protein Content

**Figure 3:**
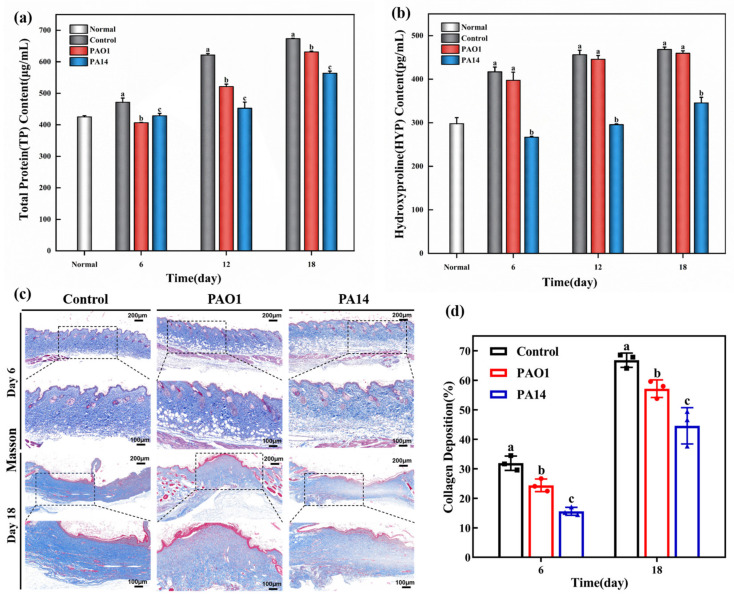
Total protein (TP) content (**a**) and hydroxyproline (HYP) content (**b**) in the wound tissues of normal, control, PAO1-infected, and PA14-infected groups on days 6, 12, and 18 postoperatively. (**c**) Masson’s staining of wound tissue of mice on days 6 and 18 postoperatively (upper magnification 5×, lower magnification 10×). (**d**) Collagen deposition in each group in (**c**). Data are expressed as mean ± standard deviation (*n* = 3). Statistically significant differences are denoted by different letters (*p* < 0.05).

Total protein (TP) levels play a critical role in the growth of granulation tissue and may reflect cellular proliferation, differentiation, and matrix synthesis within wounds [16]. Consequently, TP is a key factor influencing the success of wound healing. The TP content in the wound tissues of each group was measured using a TP assay kit, as depicted in Figure 3a. The results demonstrated a gradual increase in TP levels over time throughout the study. On day 6 post-wound modeling, TP levels in the PAO1-infected and PA14-infected groups were significantly lower than those in the uninfected control group. Specifically, the TP level in the PAO1-infected group was 406.80 ± 0.8 μg/mL, while the PA14-infected group exhibited a slightly higher level at 428.90 ± 7.5 μg/mL (*p* < 0.05). On day 12 post-wound modeling, the TP level in the control group had risen to 621.43 ± 4.85 μg/mL, marking a significant increase compared to the infected groups. Although TP levels improved in both infected groups, they remained considerably lower than those in the control group, with the PA14-infected group having the lowest TP level at 452.70 ± 19.40 μg/mL (*p* < 0.01). On day 18 post-wound modeling, a similar trend to that observed on day 12 was noted, with minimal changes in TP levels in the PA14-infected group throughout the study period. This group continued to exhibit a significant difference compared to the control group (*p* < 0.01). In conclusion, inoculation with *P. aeruginosa* impedes the synthesis of TP in granulation tissue, thereby delaying the skin’s healing process.

Hydroxyproline (HYP), an amino acid specific to collagen [17], constitutes 13.4% of collagen’s mass fraction [18], making it a key indicator of tissue collagen content. Figure 3b illustrates the variation in HYP content during the study period, which exhibited an overall increasing trend. Compared to normal skin (297.80 ± 11.35 pg/mL), HYP levels in the wound tissue of the PA14-infected group were significantly lower on days 6 and 12 post-wound modeling (*p* < 0.01). From day 6 to day 18 post-wound modeling, the PA14-infected group consistently showed the lowest HYP levels, significantly differing from those in the other groups (*p* < 0.01). In contrast, no significant difference was observed between the uninfected control group and the PAO1-infected group. These findings suggest that PA14 infection impairs HYP synthesis in wound tissue, thereby hindering collagen synthesis and accumulation and ultimately exerting an inhibitory effect on wound healing.

To facilitate a more intuitive observation and analysis of collagen distribution in wound tissues, Masson’s staining was employed. The results, presented in Figure 3c, demonstrate that the collagen fibers were stained blue. Typically, collagen deposition increases gradually over time [19]. On day 6 post-wound modeling, collagen fibers were more sparsely distributed in the two infected groups compared to the uninfected control group, with a particularly marked difference in the PA14-infected group. The control group displayed thick and densely arranged collagen fibers, while both infected groups exhibited thinner collagen deposition and incomplete collagen fiber growth, particularly evident in the PA14-infected wounds. Quantitative analysis of collagen deposition (Figure 3d) revealed that on day 18 post-wound modeling, collagen levels were significantly higher in the uninfected control group (66.81 ± 1.97%) compared to the PAO1-infected group (57.13 ± 2.44%) and the PA14-infected group (44.57 ± 5%). Statistically significant differences were observed between the control group and the infected groups (*p* < 0.05). Collagen is known to support new tissue growth and repair [20], playing a crucial role in skin injury regeneration. The reduced collagen deposition in both infected groups suggests that *P. aeruginosa* may impede the impairment repair process.

### 2.4. Effect of P. aeruginosa Infection on Wound Immune Response

**Figure 4:**
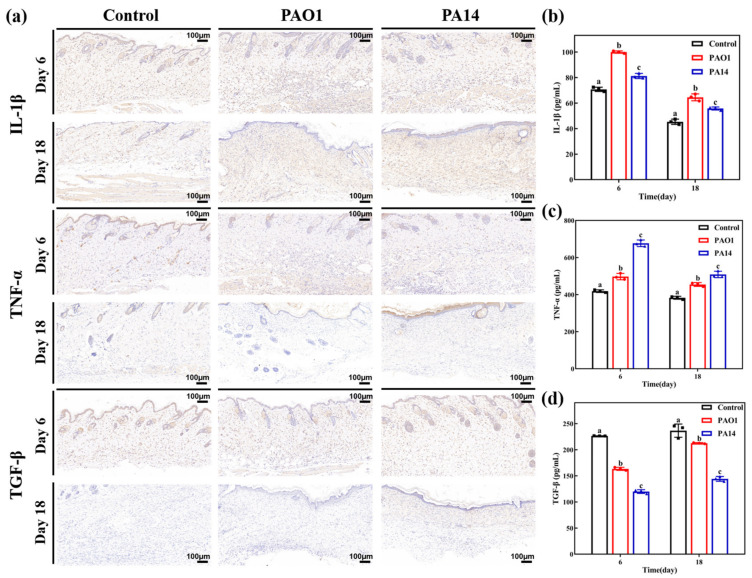
Analysis of wound tissue cytokines in each group. (**a**) Immunohistochemical staining of wound tissue cytokines IL-1β, TNF-α, and TGF-β on days 6 and 18 after surgery. (**b**) IL-1β, (**c**) TNF-α and, (**d**) TGF-β were quantitatively analyzed. All the images are magnified 10×. Data are expressed as mean ± standard deviation (*n* = 3). Statistically significant differences are denoted by different letters (*p* < 0.05).

The inflammatory response is a self-defense mechanism of the organism against external injury, involving processes such as apoptosis, proliferation, and migration. This response constitutes an immune reaction in which various cells and cytokines are engaged [21]. In the context of wound infection, IL-1β and TNF-α are characteristic pro-inflammatory cytokines, whereas transforming growth factor TGF-β plays a role in tissue repair. The expression levels of these cytokines can reflect the extent of inflammation induced by pathogen invasion. Figure 4a illustrates the results of immunohistochemical staining for the three cytokines. On day 6 post-wound modeling, wounds from the PAO1-infected and PA14-infected groups exhibited increased secretion of IL-1β and TNF-α, along with decreased levels of TGF-β compared to the uninfected control group. This suggests an excessive inflammatory response in the wounds of mice in the infected groups. On day 18 post-wound modeling, the expression levels of pro-inflammatory cytokines in the wounds of both infected groups remained elevated compared to the uninfected control group, despite a general decrease in overall inflammatory cytokine levels.

Quantitative analysis of cytokine expression (Figure 4b–d) revealed that the highest levels of IL-1β were observed in the wounds of mice from the PAO1 group on day 6 (99.97 ± 0.9 pg/mL) and day 18 (64.53 ± 2.71 pg/mL). Conversely, the highest expression levels of TNF-α were detected in the wounds of mice from the PA14 group on day 6 (676.97 ± 17.85 pg/mL) and day 18 (608.70 ± 17.41 pg/mL). In contrast, the highest expression levels of TGF-β were observed in the wounds of mice from the uninfected control group on day 6 (226.53 ± 0.25 pg/mL) and day 18 (236.57 ± 12.72 pg/mL). Statistical analysis revealed significant differences in cytokine expression levels among the wounds of the three groups of mice (*p* < 0.05). These results suggest that *P. aeruginosa* infection induces the overexpression of pro-inflammatory cytokines, which impairs collagen synthesis and neovascularization, consequently prolonging the impairment repair process.

### 2.5. Histopathologic Analysis of P. aeruginosa-Infected Wound Tissue

**Figure 5:**
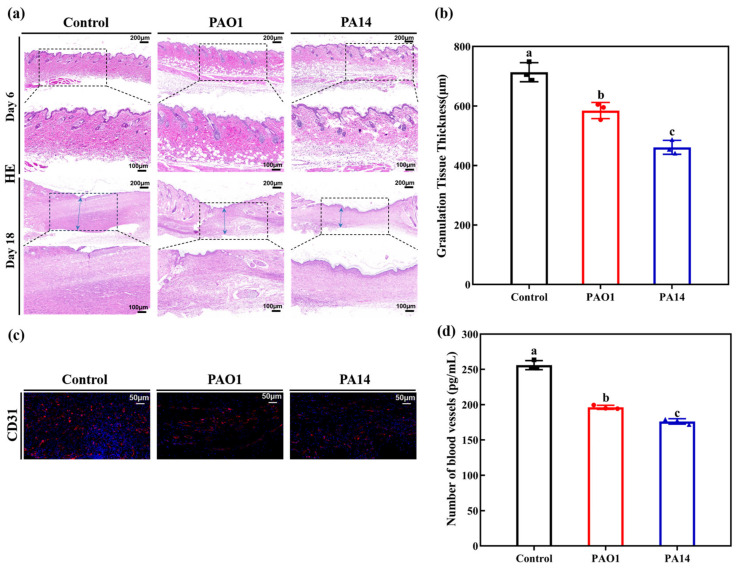
Postoperative histopathological analysis of mice in each group. (**a**) Hematoxylin and eosin (HE) staining of mouse wound tissue on postoperative days 6 and 18 (upper magnification 5×, lower magnification 10×). (**b**) Granulation tissue thickness of wound tissue in each group on day 18 in (**a**). (**c**) Immunofluorescence staining of wound tissue for CD31 (vascular biomarker) on day 18 (magnification 20×). (**d**) Quantitative analysis of CD31 in (**c**). (**a**) Blue arrows indicate granulation tissue. Data are expressed as mean ± standard deviation (*n* = 3). Statistically significant differences are denoted by different letters (*p* < 0.05).

Granulation tissue functions as a scaffold for cell migration and proliferation, while also playing a crucial role in regulating the inflammatory response. Analyzing the distribution and thickness of granulation tissue provides additional insight into the extent of damage and inflammatory response in wound tissues. Consequently, hematoxylin and eosin (HE) staining was employed to examine the wound tissues. Figure 5a illustrates that on day 6 post-wound modeling, the interstitial dermis in the two infected groups was sparser and exhibited less granulation tissue compared to the uninfected control group. This suggested a higher presence of necrotic tissue at this stage, particularly pronounced in the PA14-infected group. On day 18 post-wound modeling, granulation tissue formation had increased across all groups. The granulation tissue in the uninfected control group became thicker and denser, while that in the infected groups appeared thinner. Quantitative analysis of granulation tissue thickness (Figure 5b) corroborated the HE staining results. On day 18, the thickness of granulation tissue in the uninfected control group (713.67 ± 32.08 μm) was significantly greater than that in the PAO1-infected group (584.70 ± 27.18 μm) and the PA14-infected group (461.03 ± 23.60 μm). Statistical analysis revealed a significant difference in collagen deposition between the uninfected control group and the two infected groups (*p* < 0.05).

Neovascularization is crucial for providing adequate blood supply to wound tissues, and inadequate blood flow impairs the delivery of oxygen and nutrients to these tissues [22]. To assess neovascularization, CD31, a specific marker [23], was employed through immunofluorescence staining on day 18 post-wound modeling. The results (Figure 5c) demonstrated a significant reduction in neovascularization in both infected groups compared to the uninfected control group, with the PA14-infected group showing the lowest level of neovascularization. This finding was further validated by quantitative analysis of neovascularization using ELISA (Figure 5d).

## 3. Discussion

*P. aeruginosa* is a drug-resistant pathogen that commonly results in infected wound tissues characterized by skin erythema, pus secretion, and the rapid invasion of surrounding healthy tissues, leading to significant tissue damage [24]. Therefore, the establishment of a standardized animal model of *P. aeruginosa*-infected wounds, along with a thorough exploration of the pathogen’s pathological characteristics in wound infections, is essential for the development of effective therapeutic strategies. In this study, we inoculated adequate amounts of *P. aeruginosa* into the wound tissues of mice to assess its effects, including the bacterial impact on impairment healing, the mice’s immune response to the pathogen invasion, and the dynamic changes of *P. aeruginosa* within the wounds. Additionally, we analyzed protein content, immune responses, and pathological changes in the wound tissues, providing critical data for establishing a standardized model of *P. aeruginosa* infection in mice.

**Figure 6:**
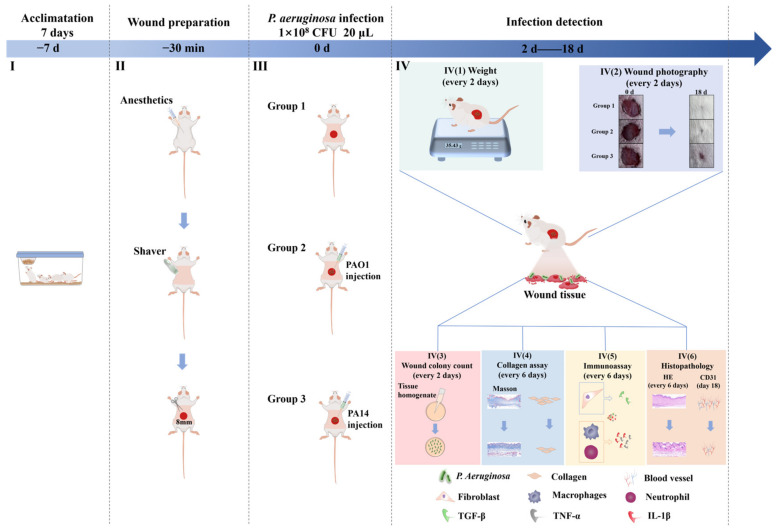
Construction procedure of a standard model of back wound in mice infected by *P. aeruginosa*.

To establish a standardized mouse wound model for *P. aeruginosa* infection, we followed the steps outlined below. It was first necessary to select an appropriate mouse strain. In this study, Kunming (KM) mice were chosen due to their stable physiological characteristics and homogeneous genetic background, which help minimize experimental variability and enhance reproducibility and reliability. Additionally, KM mice were previously utilized in a study by Fu et al. [25] investigating wound dressings, further supporting their suitability for this type of research. Secondly, during the initial phase of pathogen invasion, it was essential to house the mice temporarily for seven days (Figure 6I) to facilitate their adaptation to the new environment [26]. This acclimation period was crucial for reducing the stress associated with environmental changes and minimizing potential interference with the experimental outcomes. Ensuring that the mice were in a stable condition prior to the experiment helped mitigate the risk of experimental errors. Finally, careful control of skin injury size was necessary to accommodate a sufficient bacterial load of *P. aeruginosa*, thereby ensuring adequate infection and the validity of the experimental results. An 8 mm diameter lesion has commonly been utilized in various infection models. Xu et al. [27] created 8 mm diameter lesions on the backs of C57BL/6 mice for wound healing studies, while Liu et al. [28] similarly employed an 8 mm diameter lesion for similar research. To maintain wound consistency, this study utilized an 8 mm diameter skin biopsy punch to create a circular impression on the back of shaved mice (Figure 6II); this was followed by excising the wound with sterile surgical scissors. These measures ensured the consistency and comparability of the experiments, thereby effectively establishing a reliable mouse wound model for *P. aeruginosa* infection.

Different subtypes of *P. aeruginosa* may exhibit varying pathogenic mechanisms and levels of pathogenicity. Therefore, evaluating multiple subtypes was crucial to ensure that the standard model of *P. aeruginosa*-infected wounds was broadly applicable. In this study, we utilized the standard strains PAO1 and PA14 to construct a mouse infection model (Figure 6III). These strains are among the most commonly used standard strains for *P. aeruginosa* and possess distinct genetic backgrounds and physiological properties. By assessing the lesion extent, infection rate, and impact on the host immune response for these strains in the mouse wound infection model, we aimed to enhance the model’s credibility and representativeness. Additionally, the success of *P. aeruginosa* infection is closely linked to the concentration of the bacterial solution and the volume of inoculum. In this study, we administered 20 μL of a 1 × 10^−8^ CFU bacterial solution to ensure sufficient bacterial presence on the wound, effectively simulating an infection state. In contrast, Cheng et al. [29] inoculated mouse wounds with a concentration of only 1 × 10^−6^ CFU, indicating that an increased inoculum volume may be necessary to improve pathogen invasion success rates. It has been confirmed that both sufficient inoculation concentration and volume significantly influence the success rate of infection.

Following the establishment of a mouse wound model for *P. aeruginosa* infection, a comprehensive analysis of the condition of the infected mice was essential for assessing the model’s effectiveness. Initially, this study examined changes in mouse body weight (Figure 6IV(1)) and infection-induced symptoms such as redness, swelling, and pus exudation from the wound (Figure 6IV(2)). Pathogen invasions can lead to decreased appetite or reduced food intake in mice, which may impact their weight gain. Redness and pus exudation are characteristic signs of pathogen invasion [30] and significantly influence wound healing rates. The experimental results demonstrated that infection led to reduced weight gain, along with redness and swelling of the impairments, and significantly delayed wound healing in the mice. These findings provided preliminary confirmation of the successful establishment of the wound infection model. The observed effects could be attributed to the presence of *P. aeruginosa* in the wound, as the mouse immune system may have inadequately cleared the bacteria due to their drug resistance and ability to evade immune responses [31]. Consequently, after the initial construction of the infection model, it was essential to monitor the dynamics of *P. aeruginosa* within the wounds (Figure 6IV(3)) to evaluate the model’s stability and reproducibility. Although a general trend of gradual bacterial decrease was observed, *P. aeruginosa* persisted for an extended duration, corroborating findings from Jiang et al. [32] in their study on MRSA-infected mouse wounds.

Secondly, total protein (TP) quantifies all proteins present during the granulation phase of wound tissue growth [33]. Collagen, the predominant protein in the skin, plays a crucial role in promoting cell attachment, migration, and subsequent tissue regeneration [34]. Thus, after establishing a standardized model of *P. aeruginosa*-infected wounds, it was crucial to examine these factors (Figure 6IV(4)). Additionally, pro-inflammatory factors are molecules that promote inflammatory responses [35]; elevated levels can provoke an excessive immune reaction. To further validate the successful construction of the infection model and elucidate the infection mechanism, it was important to analyze the changes in pro-inflammatory factors induced by the infection (Figure 6IV(5)). Finally, the overexpression of pro-inflammatory factors further impedes the formation of granulation tissue and neovascularization. Granulation tissue, composed of extracellular matrix, fibroblasts, and various growth factors, is vital for the removal of necrotic tissue and the filling of tissue defects [36], serving as a precursor to neovascularization [37]. Consequently, both granulation tissue and neovascularization were analyzed in this study (Figure 6IV(6)). By comprehensively evaluating these six indicators, the efficacy of the *P. aeruginosa*-infected mouse wound model could be thoroughly assessed.

In summary, *P. aeruginosa* invasion of wounds leads to tissue damage, delays healing, and induces inflammatory responses and immune dysregulation. A standardized wound model facilitates a systematic investigation of the immune mechanisms, including cytokine responses induced by *P. aeruginosa* infection, as well as specific alterations in biological processes such as protein synthesis. An effective mouse model for *P. aeruginosa*-infected wounds should incorporate appropriate mouse strains, standardized wound preparation methods, and controlled inoculation doses to ensure reproducible and accurate results. These parameters facilitate the assessment of changes in six key indicators post-infection. This study establishes a crucial basis for the comprehensive exploration of *P. aeruginosa*-infected wounds and highlights the significance and promising application of the standardized model in infectious disease research.

## 4. Materials and Methods

### 4.1. Experimental Animals

The experiment was conducted using 72 SPF-level male Kunming (KM) mice with an initial body weight of 30 ± 5 g (age 6–8 weeks) over a period of 25 days. The mice were sourced from Shanghai JieSiJie Laboratory Animals Co., Ltd. (Shanghai, China) They were housed in standard cages with unrestricted access to distilled water and standard chow, under controlled environmental conditions (temperature: 24–26 °C, relative humidity: 30–60%, 12 h light–dark cycle). All animal experiments were performed according to the National Research Council’s Guide for the Care and Use of Laboratory Animals and approved by the Shanghai Ocean University Animal Ethics Committee (SHOU-DW-2024-110).

### 4.2. Construction of a Wound Model for P. aeruginosa Infection

Following a 7-day acclimatization period, 72 Kunming mice were randomly assigned to one of three groups: control, PAO1-infected, and PA14-infected, with each group containing 24 mice (*n* = 24). The mice were anesthetized via intraperitoneal injection of tribromoethanol (Meilunbio, Dalian, China) (30 μL/g). Following anesthesia, their dorsal fur was shaved using an electric razor, and the skin was disinfected with sterile cotton swabs soaked in 75% ethanol [38]. A skin perforation biopsy instrument [39] was then used to create a circular mark with an 8 mm diameter on the dorsal surface, after which, a wound was made using sterile scissors. For the PAO1-infected group, 20 μL of *P. aeruginosa* PAO1 (obtained from Yan’an University) bacterial suspension (1 × 10^−8^ CFU) was applied to the wound, while the PA14-infected group received 20 μL of *P. aeruginosa* PA14 (obtained from Yan’an University) bacterial suspension (1 × 10^−8^ CFU). The control group did not receive any bacterial suspension. Wound progression, the mice’s mental status, and feeding behavior were monitored every 2 days to develop a septic wound model. Wound tissues were collected from mice at predetermined postoperative time points for further analysis.

### 4.3. Macroscopic Observation of Mouse Body Weight

The control, PAO1-infected, and PA14-infected mice were weighed every two days following surgery, and the changes in body weight were systematically recorded.

### 4.4. Macroscopic Observation of Wounds and Wound Healing Rates in Mice

Postoperatively, wound images were captured and recorded every 2 days. The remaining wound length was measured using a standard scale, and the wound area was calculated with Image J version 1.8.0. The wound healing rates for each group at each time point were subsequently determined using the following formula.

Wound healing rate (%) = (A_0_ − An)/A_0_ × 100%, where A_0_ represents the initial wound area on day 0 and An denotes the remaining wound area on postoperative day n.

### 4.5. Plate Count of P. aeruginosa in Wounds

Postoperatively, 0.5 g of wound tissue was randomly collected from three mice in each group every 2 days and transferred into EP tubes. Subsequently, 4.5 mL of sterile saline was added, and the mixture was homogenized using an electric grinder for 1–2 min to produce a 1:9 tissue slurry.

The tissue homogenate was diluted to appropriate concentrations, plated onto CFC agar plates (Beijing Land Bridge Technology Co., Ltd., Beijing, China), and incubated at 37 °C for 24 h to facilitate colony counting.

### 4.6. Preparation of Skin Tissue Sections from Mouse Wound Tissues

Wound tissues were initially fixed in a 4% paraformaldehyde (PFA) (Biosharp, Beijing, China) solution and then subjected to gradient dehydration using anhydrous ethanol. Following this, tissues were embedded in paraffin [25] and cooled prior to sectioning into 3 μm thick slices. The sections were subsequently placed in an oven at 60 °C and dried overnight to finalize the preparation of the wound tissue sections.

### 4.7. Histopathological Analysis

#### 4.7.1. Hematoxylin–Eosin (HE) Staining

Paraffin sections were successively immersed in xylene I for 15 min, xylene II for 15 min, absolute ethanol I for 10 min, absolute ethanol II for 10 min, 95% ethanol for 10 min, and 85% ethanol for 10 min for gradient deparaffinization. The processed sections were stained with hematoxylin for 5 to 10 min, rinsed with water, differentiated in 1% hydrochloric acid for several seconds, and then rinsed again with water. Subsequently, the sections were stained with eosin for a few seconds, rinsed with water, and dehydrated through a series of ethanol solutions: 75% ethanol for 2 min, 85% ethanol for 2 min, absolute ethanol I for 2 min, absolute ethanol II for 2 min, and, finally, xylene for 2 min until fully transparent. After removal from xylene, the sections were air-dried and mounted with neutral gum. Finally, granulation tissue was observed using a light microscope (Nikon CI-S, Tokyo, Japan), and the thickness of the granulation tissue was measured using Image J version 1.8.0.

#### 4.7.2. Masson Staining

First, gradient deparaffinization of the tissue slices to water was performed following Section 4.7.1. Next, the slices were immersed in Bouin’s solution overnight and rinsed with water. They were then stained with prepared Weigert’s iron hematoxylin for 5–10 min and then differentiated in 1% hydrochloric acid for a few seconds, followed by washing with running water. Subsequently, an acidic fuchsin staining solution was applied for 5–10 min and the samples were then treated with phosphomolybdic acid solution for approximately 5 min. After that, the slices were placed directly in an aniline blue staining solution for counterstaining for 3–5 min. A 1% acetic acid aqueous solution was used for decolorization until the blue color was removed and then the slices were sequentially immersed in 95% ethanol, absolute ethanol, and xylene until they became transparent. Once the slices had been removed, they were mounted using neutral gum. Finally, collagen deposition was observed with an optical microscope (Nikon CI-S).

#### 4.7.3. Giemsa Staining

Equal volumes of Wright–Giemsa stain and phosphate-buffered saline were mixed to prepare the Wright–Giemsa working solution. The working solution was applied dropwise onto the paraffin sections prepared in Section 4.6 and they were allowed to sit at room temperature for 3–10 min. Afterward, the sections were rinsed with water and allowed to dry, and the growth of *Pseudomonas aeruginosa* was observed using an optical microscope (Nikon CI-S).

### 4.8. Immunohistochemistry Analysis

The tissue sections underwent graded deparaffinization to water, as described in Section 4.7.1. They were then placed in an EDTA antigen retrieval buffer and subjected to antigen retrieval in a microwave oven at medium, medium-high, and high power for 5 min each. After allowing the samples to cool naturally, the sections were washed with PBS. Subsequently, the sections were incubated in a 3% hydrogen peroxide solution at room temperature in the dark for 25 min, followed by another wash with PBS. Once slightly dried, a marker was used to draw a circle around the tissue, and goat serum was added to block the sections. After the blocking solution was removed, the primary antibody (IL-1β/TNF-α/TGF-β) was applied, and the sections were incubated overnight at 4 °C. Following this, the sections were washed with PBA buffer, and, after slight drying, the corresponding secondary antibody was added and incubated at room temperature for 50 min. The sections were washed again with PBS to eliminate any excess antibodies. DAB chromogen solution was then used for staining, which was terminated with water. Finally, the cell nuclei were counterstained with hematoxylin for 5–10 min, followed by dehydration and coverslipping. The expression of each cytokine was observed using an optical microscope (Nikon CI-S).

### 4.9. Immunofluorescence Analysis

The tissue sections underwent graded deparaffinization to water and antigen retrieval, as outlined in Section 4.8. Once slightly dried, a marker was used to draw a circle around the tissue. Triton X-100 permeabilization solution was then applied at room temperature for 30 min. Following this, a 1% BSA solution was added, and the sections were incubated for another 30 min.

Next, the sections were treated with primary and secondary antibodies according to the procedures detailed in Section 4.8. After washing with PBS, and once slightly dried, DAPI staining solution was added and the sections were incubated at room temperature in the dark for 10 min. Finally, a fluorescent quenching agent was applied and, after a subsequent wash with PBS, the sections were mounted with an anti-fluorescence quenching mounting medium. The processed sections were then examined under a fluorescence microscope (Nikon CI-S) to evaluate the state of neovascularization.

### 4.10. ELISA Experiment

Tissue samples were collected on postoperative days 6 and 18 and homogenized as outlined in Section 4.4. The processed samples were then centrifuged at 3000 rpm for 10 min, and the supernatant was collected and stored. According to the ELISA kit instructions, the concentrations of total protein (TP), hydroxyproline (HYP), cytokines (IL-1β, TNF-α, TGF-β), and neovascularization markers (CD31) were determined separately. The specific steps were as follows (Shanghai Keshun science and Technology Co., Ltd., Shanghai, China).

First, the enzyme-labeled plates were prepared by setting up the standard wells and sample wells. Then, 50 µL of standard solution was added at varying concentrations to the standard wells, and 10 µL of the test samples and 40 µL of sample diluent were sequentially added to the sample wells. Next, with the exception of the blank wells, 100 µL of HRP-labeled detection antibody was added to the remaining standard and sample wells. The plates were sealed and incubated at 37 °C for 60 min, followed by washing five times. Subsequently, 50 µL each of substrate A and substrate B was added to each well, and samples were incubated in the dark at 37 °C for 15 min. Finally, 50 µL of stop solution was added to terminate the reaction and the absorbance of each well was measured at OD450 nm.

### 4.11. Statistical Analysis

SPSS 27.0 and Origin 2022 were used for statistical analysis. Data are expressed as mean ± standard deviation (*n* = 3). Statistically significant differences are denoted by different letters (*p* < 0.05). *p* < 0.05 indicates a statistically significant difference.

## Data Availability

Data is contained within the article.
